# Adenoids

**Published:** 1909-01

**Authors:** 

**Affiliations:** Atlanta, Ga.


### ADENOIDS.

DR. RIDLEY, M. D., ATLANTA, GA.

---

In selecting the subject of adenoids for this paper, I do so with the full realization that it is one which has been discussed in one form and another in a great many medical society meetings and by men of more ability and wider experience than I have or have had. In fact, there have been few important conventions at which the subject of Adenoids have not occupied a prominent position on the program.

Realizing all this it is not my intention to bring before this meeting that which is new, so much as it is to emphasize a few points which I consider to be important in the diagnosis, symptoms and relief of this pathological condition of the nasopharynx which has been found to be such a common affection and the relief of which has been attended with such brilliant results, in the health, growth, and development, both physical and mental of those who have had them.

It has not been so very long since it was determined that adenoids could produce such a chain of symptoms as we now know are the result of this condition in the nasopharynx, in fact, it is of comparative recent years that the symptoms that we now know are the result of Adenoids were not attributed to hypertrophied oral tonsils or to some stenosis of the interior nasal passage and when the tonsils were removed or the stenosis relieved the symptoms continued the Medical Profession at that time were baffled as to the cause. So here again is progress which we men of the medical profession may justly be proud.

Before entering into the real subject of the paper, I think it would be well to freshen our memories a little on the anatomy of the parts where this condition exists.

Situated back of the posterior nares, and at the junction of the pharynx is an arched cavity to which has been given the appropriate name of the nasopharynx. This cavity is limited above by the basilar process of the occipital bone, and posteriorly by the bodies of the cervical vertibrae. It is entered anteriorly by the openings of the posterior nares and on each side by the mouth of the eustachian tubes.

It is lined by mucous membrane which is continuous with that of the posterior nares. This membrane contains numerous mucous glands and is in folds and crypts whose walls are surrounded by lymphoid tissue similar in character to that of the oral tonsils.

Between the eustachian tubes and at the highest point of the nasapharynx, is quite a mass of the lymphoid tissue known as Luschka's or the Pharyngeal tissues and it is the hypertrophy of tissue that is known as adenoids.

Adenoids then is a hypertrophy of the tissue normally present and not a new growth as is frequently thought by the laity. Adenoids may appear as tough fibrous tumors or they may be of a soft, spongy character. When composed of this form they are generally podunculated and may be likened unto a piece of cauliflower. When the brief description of the anatomy of the nasopharynx we can readily understand what a close relationship exists between Adenoids and several important structures. The symptoms and results of Adenoids are many and varied, though the most common symptom is that of mouth breathing. Situated immediately back of the posterior nares an enlargement here makes it difficult to breathe with comfort through the nose, the natural manner of breathing and as a result of the difficulty the patient breathed through the mouth. As a result of continued mouth breathing the patient is given an expression of idiocy and though he may be mentally bright he is not credited so fram his looks and facical expression.

The natural lines of the face which give to it expression or lessen or destroy, and the eyes have a dull listless appearance, the bridge of the nose is broadened and the alae are pinched and thin. By continued mouth breathing the oral tonsils become hypertrophied, the crypts and lacunae deepened, which permit an admirable nest for the growth and increase of various bacilli whose toxins when taken up by the lymphoid circulation increase the liability of tuberculosis, rheumatism, diptheria and other infectious diseases. The hard palate is often deformed and on account of the unnatural manner of breathing the lungs lack development and as a result of this the chest is badly shaped.

Again on account of the inability to breath through the nose the suckling babe is often taken from its mother's breast and placed on one or more of the many baby-foods which now flood the market, when in fact, could the baby breathe properly while nursing the milk which the Lord intended for it would be nourishment sufficient.

There are other dangers which result from mouth breathing, but time will not permit an enumeration of them. Adenoids produce a catarrhal inflammation of the membrane of the nose which is most susceptible to sudden changes in the air, causing the throwing off of an enormous amount of mucous, giving the appearance of a "cold," and then the depressing action of some of the cold tars.

One of the most serious complications of adenoids is the acute and chronic supperative inflammation of the middle ear closely related to the eustachian tubes. Adenoids throw off a discharge which is frequently loaded with infectious material and this is often forced into the mid ear through the tubes by a sudden cough, sneeze or blow of the nose and once the fire is lighted and cannot be checked until the cause is removed. Should this condition continue action there is always danger of mastoid involvement with its many dangers.

Reflex conditions which are often attributed to adenoids or malnutrition, absent-mindedness, bed-wetting, night terrors, general nervousness and many others. Chronic deafness and infection of the frontal sinus can often be attributed to this condition. The early recognition of adenoids is imperative for the future welfare of the patient, and for that reason it becomes the duty of the family doctor to be aware of the symptoms and dangers of adenoids and take the necessary steps to remove them before the complications have begun. I claim that the vast majority of the cases of adenoids develop in childhood and when found in adults they have existed since that time. In my opinion adenoids like all lymphoid tissue has a tendency and does atrophy in time, another reason why I advocate their early removal is on account of the permanent damage they may do before they atrophy.

The diagnosis of adenoids can frequently be made from a chain of these symptoms, when whoever this cannot be done, the nasal rhiniscope or the index finger introduced through the mouth into the nasapharynx readily reveals adenoids when they are present.

The latter method is the most reliable of all others for when once adenoids are felt it is rarely a mistake is made in the diagnosis. The condition most frequently mistaken for adenoids in this manner is a chronic catarrhal inflammation of the nas-pharynx, when the mucous membrane is thickened and the crypts stand out more prominently than normal and when this condition is interferred with by surgical proecdure more harm is done than adenoids would have done, had they been present, as it destroys the normal mucous glands and replaces it with a mass of acatricial tissue.

The treatment for the relief of adenoids can be described in a very few words. Operative procedure for their removal is the only treatment I consider.

For this operation as in all operations, the patient should be prepared by giving a laxative the night before, and nothing to eat within four hours of the operation. A general anaesthetic should by all means be used when not contra indicated and this pushed to completely narcosis where the pharyngial muscles will be a state of relaxation. The choice of the anaesthetic is left to the experience and choice of the anaesthetist.

I know that in giving anaesthetic a great many operators differ with me, but I claim the operation cannot be done properly with a struggling patient. As soon as the instrument is introduced in a sensative larynx the pharyngeal muscles close over it and limits its use to such an extent that if force be used the soft structures of the throat is often injured as is also the orifice to the eustachian tubes. The horror and pain of such an operation where the hemorrhage is as free as it is in this, often produces such a shock to the sensitive nervous system as to impair it permenently.

The result of such a procedure, I saw in one of our medical students, who was operated upon in this manner, who consulted me about a sensitive spot in his pharynx which gave him a great deal of pain in swallowing. Upon examination I found and area about 1-8 inch in diameter of denuded surface which exposed a part of one of the cervical vertibrae. This gentleman would not have had this to happen, had he been given an anaesthetic.

The ideal instrument to use for this operation is Gottstein Curette in some of its siesz and the finger nail of the index finger. I think with these instruments the operation can be performed thoroughly and satisfactorily.

I do not mean that you understand that I use the finger nail to remove the bulk of the hypertrophy, but it is simply used to clear out the fossae of Rosenmuller, a most important step in the operation. After the Curette has been thoroughly used first. I can not see how any one can claim to do this operation with their finger nail alone unless it is that the man who operates on the patient the second time does not inform him of the fact.

The position of the patient for operation should either be the Trendelenburg or recumbent positions, with the head over the end of the table or couch. As soon as the operation is completed the patient should be turned so as to permit the hemorrhage from being drawn into the larynx.

To keep the mouth open during the operation, the mouth gag is of course used.

The after treatment consists of a simple diet, for a few days, and an ordinary spray for cleanliness and lessen chances of infection and frequent blowing of the nostrils first one side and then the other.

And now, gentlemen, in conclusion, I wish to add that I have not presented this paper with any idea of having you believe that I am a literary genius, but merely to bring out a few points which I consider to be important in the diagnosis and treatment of a most common and frequent condition, adenoids.

The important points I wish to emphasize are:

1. The early diagnosis and treatment of adenoids.

2. The absolute necessity of using a general anaesthetic.

3. And thorough removal of all hypertrophied tissue by the use of the Curette and finger.

4. Care in not wounding the eustachian tubes and at the same time clearing the fossae of Rosenmuller from all the adenoid tissue.

